# Restorative Effects of Multi-Sensory Perception in Urban Green Space: A Case Study of Urban Park in Guangzhou, China

**DOI:** 10.3390/ijerph16244943

**Published:** 2019-12-06

**Authors:** Tianyao Zhang, Jiahui Liu, Hongyang Li

**Affiliations:** 1 Research Associate, School of Geography, South China Normal University, Guangzhou 510641, China; zhangtianyao@m.scnu.edu.cn; 2 School of Geography, South China Normal University, Guangzhou 510641, China; 20152600104@m.scnu.edu.cn; 3 Associate Professor, School of Civil Engineering and Transportation, South China University of Technology, Guangzhou 510641, China

**Keywords:** visual sensation, auditory sensation, tactical sensation, mental restoration, health-related behaviors, emotional response, urban green space

## Abstract

Urban green space is believed as a beneficial landscape for mental restoration in the urban settings. This study aims to examine the restorative quality of the urban green space from a multi-sensory perspective, focusing on both direct and indirect connections between visual, auditory, and tactile sensations and mental restoration. Two hundred and fifty park users of Tianhe Park in Guangzhou, China, were surveyed. Data were collected regarding their three types of sensations, i.e., the perceived mental restoration, health-related behavioral activities and emotional responses in the urban park. As a result, visual and auditory sensation were found to be linked with mental restoration directly and indirectly, while the tactile sensation was only associated with mental restoration indirectly; and health-related behaviors and emotional response mediated the relations between the three sensations and mental restoration significantly. It is concluded that the greater effects of auditory sensation and the under-studied potential effects of tactile sensation on mental restoration should be more emphasized in the future design of urban park. This is expected to contribute to the high restorative quality of the urban green space and promote public health.

## 1. Introduction

Increasing attention has been paid to the restorative landscape associated with public health during the past decade, acknowledging the contribution of an appealing landscape to health and wellbeing [1]. The way of landscape being perceived, experienced and used as a resource for health and health-related behaviors, however, remains an open question [2]. Within the urbanized societies, urban green space has been regarded as a typical landscape for health promotion due to its independent salutogenic effects [3,4]. Substantial evidence has suggested that urban green space could generate great psychological and physiological benefits by promoting green exercises, reducing blood pressure and stress level [5], restoring mental fatigue [6], and restoring depleted emotional and cognitive resources [7]. Studies on mental restoration of urban green space in relation to health often highlight perceived sensory dimensions of the landscape [3], of particular note is that landscape is experienced in multisensory manner with sensory coordination [2,3], indicating that the perception is a holistic process integrating information from various senses such as sight and hearing [8]. However, most extant studies focus primarily on the visual sensation, which has been regarded as the most important sense when visiting urban green space, leaving out the effects of other senses in urban green space on mental restoration, such as auditory and tactile sensations [3,9]. It is thus necessary to explore the restorative effects of urban green space holistically from a multi-sensory perspective. Therefore, this study examines both direct and indirect effects of visual, auditory, and tactile sensory on mental restoration, regarding users’ behaviors occurred in urban green space and their emotional response as potential mediators, aiming to explain the restorative mechanisms of urban green space from a multisensory perspective. 

The paper is organized as follows. Section 2 consists of a summary of previous work on the restorative effects and multi-sensory characteristics of urban green space. Section 3 introduces the research approach, including data collection, variable measurements, and data analysis strategy. In Section 4, we describe the characteristics of mental restoration and multi-sensory perception in urban park, of which associations and its underlying mechanism are explained in the Section 5. Conclusions, limitations and directions for future research are given at the end of the paper. 

## 2. Restorative Effect of Urban Green Space: Characteristics and Mechanisms 

Restorative environment refers to an environment beneficial to psychological and physical health through reducing stress and various negative emotions, decreasing mental fatigue, improving attention, facilitating disease recovery, etc. [10,11]. Stemming from the attention restoration theory (ART), four dimensions of restorative environment are proposed: (1) Being away, referring to the elements that allow individuals to distance themselves physically or psychologically from the things that require their directed attention; (2) extent, referring to the richness and coherency in terms of individual exploration to the whole world, including enough to see, experience, and think about; (3) fascination, referring to the attractive characteristics that attract people’s attention, which guarantees that directed attention can rest; (4) compatibility, referring to the fitness between the environment and people’s purposes and inclinations [12]. Meanwhile, stress recovery theory (SRT) provides additional theoretical foundations for understanding the mechanisms of restorative environment [13]. Comparatively, the ART focuses on the attentional restoration from depletion of cognitive resources based on about four restorative components [12], while the SRT highlights the way of nature elements supporting affective recovery emotionally and physiologically from stress and daily hassles [7,14,15]. 

Based on these fundamental theories, a bunch of studies make efforts on the exploration of the restorative effect of natural experience, the key influential factors of mental restoration, as well as their underlying mechanisms within different local contexts [13]. Numerous studies compare the restorative effects of exposure to natural components against built components in laboratory and field settings [16], leading to a natural-built dichotomy in prevalent restorative environment research [15]. It has been acknowledged that natural environment consistently has more significant restorative effects than simulated natural environment, urban built environment, recreational environment or urban nightscape on human psychological process [17,18,19]. Nature elements associated with mental restoration in urban green environments are usually studied on the basis of the classification of perceived sensory dimensions (PSDs), comprised of eight aspects of natural preferences: Serene (silent and calm), nature (wild and untouched), rich in species, space (spacious and free), refuge (safe, benches and play equipment), culture (decorated with fountains and ornamental plants), prospect (flat and well-cut grass surfaces and vistas), and social (entertainment and restaurants) [1,3]. The significant associations between the PSDs and the perceived restorative potential of an environment are further explored by some studies within specific local contexts [1].

An expanding body of research focus on the links between landscape and health [9] paying particularly attention on the effects of urban green spaces on public health within the rapid urbanization context [20]. Studies have found positive mental and physiological effects of urban nature on human health and wellbeing [20,21]. Urban green space has been regarded as valuate outdoor resource where people can enjoy nature in the high-density urban environments [22], providing the restorative effects such as reducing stress and mental fatigue, smoothing mood, and preventing depression [15]. For instance, viewing the natural elements (e.g., vegetation and water) could ameliorate stress and benefit for patients’ recovery [9]; visiting and exercising in urban parks could release stressfulness and headache and improve the psychological balance greatly [23]. Thus, natural elements play greatly important roles in terms of improving human physical and mental health and nurturing the overall happiness [24,25]. Urban green space can improve health by providing ecological products and services, promoting physical exercise, and enhancing social capital [26]. Socio-ecological models suggest that there are three broad groups of mediators between green space and health, i.e., improved perceptions of living environment, aesthetic pleasure and relaxation from viewing green space, and use of greenspace (e.g., relaxation activities, physical activities, interaction with wildlife and nature, and social interactions) [27]. Extant efforts to explain the associations between green space and health suggest five mediators or pathways, including: Improved environmental quality, physical activity, social interaction, direct restoration via psycho–neuro–endocrine pathways, and emotional response/experience [28]. Therefore, the mediation of emotional responses and behavioral activities (including social activities and physical activities) should be highlighted in the exploration of the mental restorative effects of urban green space [27,29,30]. 

Environmental perception is multi-sensory, including the five senses or more, which could be categorized into distance sense (vision, hearing, and smell) and nearness senses (tactile and touch) [31] (p. 41). Multiple sensations work in concert to give people a full experience and understanding of their surrounding environment [31,32]. Although vision has been regarded as the most important sense when visiting urban green space, the importance of other senses should not be neglected, particularly regarding the links between the usage of urban green space and people’s health and wellbeing [3]. Besides of the large body of studies on the sense of vision and the evaluation of urban green space, another group of research emphasizes that it is the experiences associated with urban green space that is the authentic reason of people’s going out into a landscape [3]. The sensory–somatic modalities are thus proposed: “Information is processed via people’s muscles, inner organs, etc., indicating that sensations and images from the environment work as a catalyst, which is utmost importance to mental restoration” [3]. Sensory dimensions of urban green space have been regarded as direct and real feelings formed by the five senses in synergy [33], comprised of behavioral activities carrying sensory and perceptual components that lead to emotional changes [34]. To summarize, the comprehensive environmental perception is thus formed by the multiple sensations including sight, smell, taste, sound, and touch, and the emotional responses and behavioral activities occurred in urban green space are of great importance in the evaluation of the mental restoration of urban green space. 

The multi-sensory theory was originally developed to design sensory parks for specific people, enhancing multi-sensory perception and stimulation [33,35]. In practice, design of sensory parks for treating certain disease (such as the therapy of dementia) has attracted researchers’ attention from the late 1980s, when both visual and auditory sensations were considered in the provision of facilities to satisfy users’ needs [35,36]. Focusing on users’ multiple sensory dimensions, sensory parks emphasize the stimulation of various senses, communication with others, and positive emotional responses [37,38]. Multi-sensory perception in a rehabilitation garden is closely related to the users’ sense of existence, emotions, and physiology, and it could establish a wordless “communication”, which is beneficial to realize an extended emotional and existential awareness, and increase the mental restorative effect accordingly [39]. Thus, most extant empirical studies on multi-sensory perception and its recovery effect focus on the garden therapy, while the multi-sensory dimensions of the wider scale of urban green space and its mental restoration effect is still under-studied [1,40].

**Figure 1:**
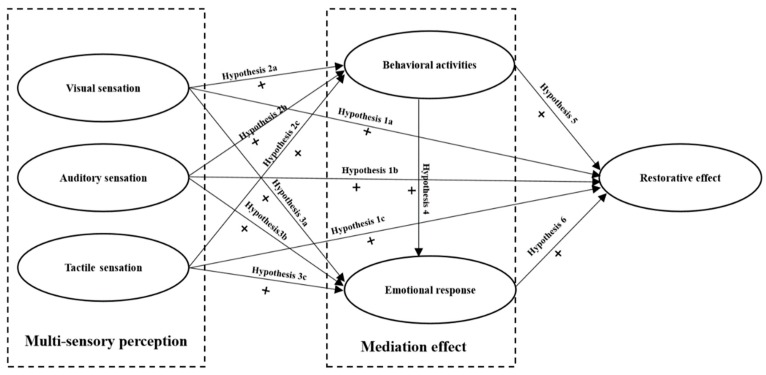
Theoretical-indicated framework of multi-sensory perception and restorative effects of urban parks.

To provide evidence for plan and design of urban park with restorative qualities, this study aims to examine the relationship between users’ multi-sensory perception and the perceived mental restoration of urban parks in China through investigating the restorative effects of urban parks from the lens of perceived multi-sensory perspective. The hypothesis is set as that: The multi-sensory perception in urban parks could influence user’s perceived mental restoration both directly and indirectly. Emotional responses and behavioral activities occurred in the park (physical activity and social interaction) could mediate between multi-sensory perception and people’s perceived mental restoration in urban parks. A theoretical-indicated framework is thus developed: individual’s visual, auditory and tactile sensation in urban parks affect the restorative effects of urban parks directly and through the mediation of emotional response and behavioral activities (Figure 1). The detailed hypotheses are proposed:

**Hypothesis 1** **(H1).** *The multi-sensory dimensions of urban parks have significant positive impacts on the restorative effects.*

**Hypothesis 1a** **(H1a).** *The visual sensation of urban parks has significant positive impacts on the restorative effects.*

**Hypothesis 1b** **(H1b).** *The auditory sensation of urban parks has significant positive impacts on the restorative effects.*

**Hypothesis 1c** **(H1c).** *The tactile sensation of urban parks has significant positive impacts on the restorative effects.*

**Hypothesis 2** **(H2).** *The multi-sensory dimensions of urban parks have significant positive impacts on residents’ behavioral activities.*

**Hypothesis 2a** **(H2a).** *The visual sensation has significant positive impacts on residents’ behavioral activities.*

**Hypothesis 2b** **(H2b).** *The auditory sensation has significant positive impacts on residents’ behavioral activities.*

**Hypothesis 2c** **(H2c).** *The tactile sensation has significant positive impacts on residents’ behavioral activities.*

**Hypothesis 3** **(H3).** *The multi-sensory dimensions of urban parks have significant positive impacts on residents’ emotional responses.*

**Hypothesis 3a** **(H3a).** *The visual sensation has significant positive impacts on the emotional responses of residents.*

**Hypothesis 3b** **(H3b).** *The auditory sensation has significant positive impacts on the emotional responses of residents.*

**Hypothesis 3c** **(H3c).** *The tactile sensation has significant positive impacts on the emotional responses of residents.*

**Hypothesis 4** **(H4).** *The residents’ behavioral activities have significant positive impacts on the emotional responses of residents.*

**Hypothesis 5** **(H5).** *The residents’ behavioral activities have significant positive impacts on the restorative effects.*

**Hypothesis 6** **(H6).** *The emotional responses of residents have significant positive impacts on the restorative effects.*

## 3. Methodology

### 3.1. Case Selection and Data Collection 

**Figure 2:**
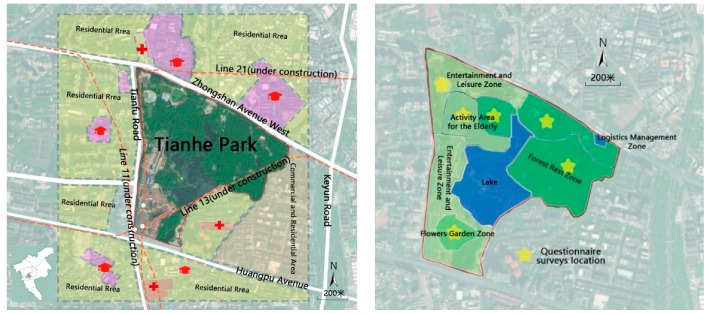
The location of Tianhe Park, Guangzhou.

Tianhe Park in Guangzhou is selected as the typical case for this study. Located in the center of Tianhe district, Tianhe Park serves as a central park on urban level, attracting urban residents from the whole city (Figure 2). It enjoys convenient transportation conditions, surrounded by two urban arterial roads and three subway lines (Line 11, 13, and 21). In addition, it is neighbored by residential neighborhoods, universities, office space and public facilities (such as hospitals), providing an accessible urban green space for various population. Designed as a comprehensive urban park, Tianhe Park covers a total area of 70.7 hm^2^, including water area (10 hm^2^) and land area (60.7 hm^2^). In terms of the landscape element, Tianhe Park is comprised by various natural elements such as trees, shrub, lawn, lakes, and hills, resulting in a green coverage ratio as high as 90%. Functionally, it is designed into five zones: Flowers garden zone, entertainment and leisure zone, activity area for the elderly, forest rest zone, and logistics management zone. 

We carried out questionnaire surveys during 11 March 2019 and 16th March 2019 to gain the information on the perceived multi-sensory dimensions of Tianhe Park and users’ perceived mental restoration. Two modes of survey were conducted to improve the representativeness of our samples: On-site face-to-face survey and online survey. The on-site survey was conducted on both weekdays and weekend from 7 a.m. to 7 p.m., using a random sampling method at five different spots in the five zones, respectively. The online questionnaire was supplement used to enlarge the range of target population, including those who have been to the Tianhe Park from time to time but didn’t come to the park during the on-site survey period. Finally, 174 on-site questionnaires and 85 online ones were collected. Excluding the invalided respondents such as the same responses for each single item and incomplete responses, there were 250 valid respondents left in this study.

### 3.2. Measurements

#### 3.2.1. Restorative Effect

Based on the ART and referring to existing measurements of restorativeness, a five-point Likert scale comprised of six items was developed to measure the perceived mental restoration: (1) *I feel relieved from physical pain and discomfort*; (2) *I feel relieved from anxiety, depression and stress*; (3) *My attention is enhanced*; (4) *My health is good and my level of activity is improving*; (5) *I feel loneliness is reduced*; and (6) *I feel my quality of sleep is improved.* Respondents were asked to rate 1–5 to indicate the level of agreement with above statements. 

#### 3.2.2. Multi-Sensory Perception

Perceived multi-sensory dimensions of the urban park were investigated by a five-point Likert scale (from strongly disagree to strongly agree), focusing on users’ visual sensation, auditory sensation and tactile sensation particularly. Visual perception was measured by six items: Variety of plants (V1), richness of plants’ color (V2), plant light and shadow mottle (V3), nice road texture (V4), rich terrain and wide view (V5), the water is highly ornamental (V6). Auditory perception was measured by five items: Natural sounds (A1), sweet background music (A2), happy people sounds (singing or playing instruments) (A3), quiet space (background city) (A4), and no traffic noise (A5). Tactile perception was investigated by four items: the road material is comfortable and the foot feels good (T1), strong hydrophilic (T2), the seat is comfortable for sitting (T3), and there is comfortable grass for flat lay (T4) [3]. 

#### 3.2.3. Emotional Response and Behaviors in Urban Park

Two mediators between multi-sensory perception and mental restoration, emotional responses and behavioral activities, were investigated by five-point Likert scales, respectively. First, four types of emotional responses were evaluated: comfort, happiness, sense of belonging, and attraction. The intensity of these feelings was measured by five degrees, ranging from very strong, strong, neutral, weak, to very weak. Second, six types of behavioral activities that often occur in the park was investigated, including the activities of relaxation and thinking, social interaction, fitness exercise, walking, art activity, and family activities. The frequency of these activities was evaluated by five levels, ranging from always, often, sometimes, occasionally, and never. 

#### 3.2.4. Individual Correlates

Socio-demographic characteristics and visiting habits were investigated as the individual correlates since they are regarded as the potential confounders between environmental perception and mental restoration. These individual correlates include: gender, age, general health status, companion, duration of stay, and attitude on the importance of urban park for stress releasing. 

Reliability and validity of above variables are reported in Table 1. The Cronbach’s alpha of the total variables was 0.931. The Cronbach’s alpha of each set of those indicators was above 0.7, and the combination reliability was also over 0.6. It showed that the observed variables of each latent variable were better designed, and the reliability of the questionnaire was high. At the same time the KMO value was 0.921, which was greater than 0.7. The *p* value was (0.000) less than 0.001. The extracted value (AVE) of each latent variable was almost above 0.45, which was close to or greater than the critical criterion (0.5), indicating that the selected observed variable had a certain correlation. 

### 3.3. Statistical Analysis 

To examine the theoretical-indicated hypotheses proposed in Section 2, structural equation model was performed to examine the underlying pathways between multi-sensory perceptions and mental restoration. A path analysis was used to assess the theoretically-indicated interplay between all factors. Four theoretical pathways were proposed: (a) Multi-sensory perception -> restorative effect; (b) multi-sensory perception -> behavioral activities -> restorative effect; (c) multi-sensory perception -> emotional response -> restorative effect; and (d) multi-sensory perception -> behavioral activities -> emotional response -> restorative effect. To explain the mediation effect of behavioral activities and emotional response, the bias-corrected deviation correction method was used to estimate the total, direct and indirect effects of the multi-sensory perception on the restorative effects. The mediation effect analysis was performed under the condition of repeated sampling of 5000 times using Bootstrapping. The statistical analysis was conducted by the software Amos 21, and coefficient and standard error (SE) for all paths were reported.

## 4. Results

### 4.1. Descriptive Analysis

Descriptive statistics of the samples are presented in Table 2 and Table 3. For the socio-demographic characteristics, more than one half of the respondents (58.00%) were female and most respondents were 18–30 years old. Most respondents (78.40%) reported good health, with no one reported poor health, indicating that users of the park were generally in a good health condition. For the vising habits, almost one-fourth of the respondents (24.12%) went to the park with friends, around one-fifth of the respondents’ companion was lover (20.95%) and another one-fifth of the respondents went the park by themselves (21.30%). Similar proportions of the respondents spent 20 min to 1 h (34%) and 1 to 2 h (36%) in the park, respectively, while only 8.4% of respondents spent less than 20 min in the park per time. All respondents believed that the park was influential for stress relieving except those who had no idea. 

For the restorative effect, the average scores of R1, R2, and R5 ranged from neutral to agree (3.76–3.96) in the five-point scale, and the average scores of R3, R4, and R6 ranged from agree to strongly agree (4.04–4.12), indicating positive restorative effects of the park, especially in terms of the promotion of attention recovery and mental wellbeing, physical health and activity level, and sleep quality. For visual sensation, mean scores of V1, V2, V3, V4, V5, and V6 ranged from agree to strongly agree (4.00–4.24), with V6 was lower than 4 in the five-point scale, indicating good visual sensation of the park except for the ornamental value of water. Similarly, among the five indicators of auditory sensation, only one item’s (A2) average score was lower than 4 in the 5-point scale, suggesting good auditory sensation except for the background music. For tactile sensation, the mean scores of hydrophilic (T2) and comfortability of the grass (T4) were lower than 4, indicating that the relatively poorer perception on these two aspects than on the road material (T1) and seat quality (T3). 

For behavioral activities, the frequency of literary activities (B4) was the lowest, between occasionally and sometimes (mean = 2.68), to contrast, the frequency of other activities was all between sometimes and often (mean scores ranged from 3.06 to 3.84) in the five-point scale ranging from always to never. For the emotional response, respondents tended to report strong to very strong perceptions of comfort, pleasant, and attraction (4.20–4.27), while the average score of sense of belonging was between neutral and strong (3.98), indicating less intensity of the feel of belonging. 

### 4.2. Path Analysis of Multi-Sensory Perception and Restorative Effect 

Normality test on all observed variables indicate the normal distribution of the data set, and the structural equation model produced acceptable fit indices as shown in Table 4. The values of GFI, CFI, AGFI, TLI, and CFI in the model were slightly lower than the ideal values, but the overall fitting degree is acceptable for this data [41,42,43]. The results of confirmatory factor analysis suggested that all scales used in this study formed adequate measurement models, and the construct validity of the measures was confirmed. 

The factor loading of the latent variables on all indicators were relatively high, ranging from 0.41 to 0.81. For visual sensation, the loading of rich in plant species, the standardized path coefficients of plant colors, plant light and mottled, road texture, terrain, and water body were respectively 0.68, 0.68, 0.60, 0.65, 0.59, and 0.60. For auditory sensation, the standardized path coefficients of the natural sound, background music, singing and dancing, quiet space, and traffic noise were 0.73, 0.52, 0.48, 0.63, and 0.63, respectively. For tactile sensation, the standardized path coefficients of the material of the road, the foot feeling, the hydrophilicity, the seat, and the grass were 0.63, 0.73, 0.73, and 0.68, respectively. (Table 5)

**Figure 3:**
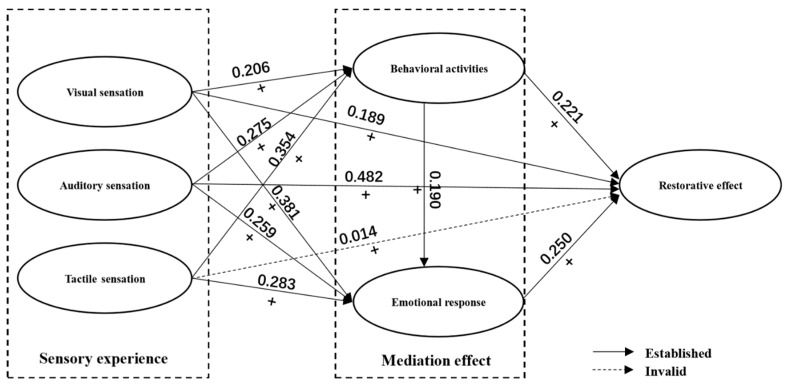
Structural equation model path coefficient.

Standardized coefficients and their statistical significance obtained from the structural equation model were presented in Table 6. Results indicated that 11 assumptions were established except for the Hypothesis 1c of the effects of tactile sensation on restorative effect. Specifically, both visual and auditory sensations were positively associated with restorative effect significantly. There was however no direct association was found between tactile sensation and restorative effect. All three types of sensory perception were positively associated with the frequency of behavioral activities and the intensity of positive emotional responses significantly. The frequency of behavioral activities was positively associated with restorative effect and the intensity of emotional response. The intensity of positive emotional response could contribute to the restorative effect significantly. Furthermore, the standardized coefficients of the visual sensation, auditory sensation, tactile sensation, frequency of behavioral activities, and emotional responses to the restorative effects were 0.189, 0.482, 0.014, 0.221, and 0.250, respectively, implying that the auditory sensation had the greatest effect on the restorative effect (Figure 3).

For the mediation effects of behavioral activities and emotional response, results were presented Table 7. For the associations between visual sensation and restorative effect, the estimates of total effects, direct effects and indirect effects did not contain 0 in the 95% confidence interval, suggesting that the visual sensation had an incomplete mediating effect on the restorative effect. In addition, there was an incomplete mediating effect in the associations between auditory sensation and restorative effect as well. However, the direct effect of the tactile sensation on the restorative effect included 0 in the 95% confidence interval, suggesting a complete mediating effect on the restorative effect. The tactile sensation thus affected restorative effect indirectly through influencing residents’ behavioral activities and emotional responses. The direct and indirect effect of visual sensation on restorative effect account for 56% and 44%, respectively, while those of auditory sensation on restorative effect 78% and 22%, respectively. Comparatively, the auditory sensation had more effects than the visual and tactile sensation on the mental restoration; and the direct effects of the visual sensation and the auditory sensation were much stronger than their indirect effects.

## 5. Discussion

Based on the primary data from the place-based survey of Tianhe Park in Guangzhou, this study examined the link between multi-sensory perception and the restorative effect of the urban green space. Urban park users’ multi-sensory perception plays a significant role in their behavioral activities occurred in the urban park, emotional responses generated in the urban park, and the mentally restorative experiences of the urban park. For the underlying pathways, specifically, the visual sensation and the auditory sensation had both direct and indirect links with mental restoration, while the tactile sensation only affected mental restoration indirectly. In addition, mediations of the frequency of behavioral activities and the intensity of emotional response between multi-sensory perception and mental restoration were also confirmed. This study thus could enrich the evidence of the health implication of the landscape perception in a multisensory manner to some extent [2], particularly by highlighting the restorative effects of the visual, auditory, and tactile sensations in the urban park.

Regarding the visual perception of the urban park, this study consistently ascertains the positive effects of viewing the natural elements (i.e., terrain, plants, light, road, and waters) of the park on the level of restorative effects directly and through the mediating process. This supports the established knowledge that viewing vegetation, water, and other naturel elements could ameliorate stress, promote more positive moods and feelings, induce behavioral changes that could improve mental and physical health, and be beneficial for recovery from illness [9,44]. For instance, Ulrich et al. [14] point out that looking at a natural landscape could unconsciously and immediately release emotional reactions that contributing to stress recovery.

Focusing on the perception of sounds in the park, we confirmed the significant role of urban park users’ auditory sensation in their restorative experience of the urban park. First, the comfort auditory sensation generated in the urban park could increase park users’ mental restoration directly. We propose that natural sounds, ‘happy people’ sounds such as singing and music, quiet background city, and less traffic noise could contribute to a pleasant soundscape environment that nurtures better mental restoration. This finding echoes the established knowledge that accessing to green areas could lower the noise annoyance and stress-related psychosocial symptoms and improve relaxation and sleeps to some extent [45]. Second, park users’ auditory sensation could influence mental restoration through mediating the occurrence of health-related behavioral activities and positive emotions. Better auditory sensation is believed to be associated with more frequent occurrence of the health-related behaviors and more positive emotional response, leading to a higher level of mental restoration for the park users, which is consistent with the previous finding that people’s daily behavior such as the use of space outdoors and the frequency with which they visit urban parks could mediate the restorative level of green space [45,46,47]. This supports the literature that the activities that an individual carry out are important to his or her perception of sounds, interpretation on the sounds and preference to sounds [47,48,49].

For the association between tactile sensation and mental restoration, this study argues that the perception of touching may determine the restorative quality of the urban park through influencing the health-related activities and the resultant emotions. Few existing evidences could be found in previous studies, which may be due to the absence of the direct link between tactile sensation and mental restoration as suggested by this study. However, the tactile sensation formulated by the contact with natural elements (i.e., lawn and waters) and the use of facilities (i.e., seats and footpath) could determine the health-beneficial activities that occurred in the urban park and evocate emotional responses, affecting the restorative effects accordingly. This study thus particularly highlights the potential role of tactile sensation of the urban park on the restorative effects.

Therefore, based on the restorative effect of multi-sensory perception, health-related behaviors and emotional responses of the urban park found in this study, we provide more local evidences supporting the argument that “the experience generated in a place is just as important as the presence of certain physical aspects in making a place restorative to the individual” [47,49]. For the multi-sensory perception of the urban park, we surprisingly found that the auditory sensation has the largest effects on the mental restoration across the three sensations. Although some scholars emphasize that health benefits of natural experience depend on noticing and observing the natural elements rather than performing activities in nature [50] (pp. 125–133), this study supplements the contributions of multi-sensory perception and health-related behaviors to mental restoration in the urban park within Chinese context. For the underlying mechanisms of multi-sensory perception affecting mental restoration, behavioral, and emotional ways work simultaneously in general. On one hand, various studies have acknowledged the positive effects of physical activity and social interactions on mental and physical recovery of general population, as well as their mediating roles between urban green space and public health [2,51,52]. On the other hand, emotional response has been usually regarded as an important element linking the green space and restrorativeness: the green landscape could induce positive feelings such as interest, cheerfulness, and calmness, which could replace negative feelings and thoughts [53,54]. Thus, this study echoes the existing arguments by arguing that the frequency of health-related activities in the urban park and the intensity of the emotional response serve as the mediators between individual multi-sensory perceptions and mental restoration.

## 6. Conclusions

The study identified the mental restorative effects of multi-sensory perceptions of the urban park, and examined the mediating effects of health-related behaviors carried out in the park and the resultant emotional responses. We ascertained the direct association between the visual and auditory sensations and mental restoration, the indirect association between tactile sensation and mental restoration, as well as the mediation of behavioral activities and emotional responses between the three multi-sensory perceptions and mental restoration. In comparison, it is particularly found that auditory sensation has relatively greater contribution to mental restoration than the other two sensations—calling for further explanations in future studies. Operationally, the multi-sensory perception perspective should be considered seriously when designing urban green space, particularly in terms of the direct restorative effects of auditory and visual sensations, and the requirements of users’ health-related behaviors and emotions.

There have been advances in our understanding of the associations between multi-sensory perception and mental restoration. Nevertheless, limitations of this study should be admitted: Due to the numerus variables included in the research design, only the main effects of the three sensations on mental restoration were focused on, that is, the potential interactions across the sensations and their potential effects on mental restoration have not been observed. Identifying the effects of mutual interactions of multi-sensory perceptions on the restorative quality of urban green space is one of the major necessity research of the future.

## Figures and Tables

**Table 1:** Reliability and validity analysis.

Latent Variable	Observation Variable	α	CR	AVE
Visual sensation	6	0.798	0.80	0.41
Auditory sensation	5	0.741	0.72	0.46
Tactile sensation	4	0.780	0.79	0.48
Behavioral activities	6	0.844	0.82	0.44
Emotional response	4	0.834	0.80	0.51
Restorative effect	6	0.883	0.85	0.49
Total	31	0.931		

**Table 2:** Descriptive statistics of the sample.

	Classification	Number of People	Proportion (%)
Gender	Male	105	42
	Female	145	58
Age	Under 18	12	4.8
	18–30	104	41.6
	31–45	55	22
	45–60	37	14.8
	60 or older	42	16.8
Who do you like to come to the park?	Child/Children	99	17.43
	Lover	119	20.95
	Friends	137	24.12
	Parents	92	16.2
	On one’s own	121	21.3
Time spent in the park	20 min or less	21	8.4
	20 min–1 h	85	34
	1 h–2 h	90	36
	More than 2 h	51	21.6
General health status	Very good	67	26.8
	Good	129	51.6
	General	54	21.6
	Poor	0	0
	Very poor	0	0
The importance of stress relief of the park	Very important	79	31.6
	Important	130	52
	General	38	15.2
	Unimportant	0	0
	Unclear	3	1.2

**Table 3:** Descriptive analyses of perceived mental restoration, multi-sensory perception, behavioral activities and emotional responses in the urban park (N = 250).

Items	Mean	Standard Deviation
**Restorative effect**		
R1 Physical pain, not comfort	3.76	0.909
R2 Anxiety, depression, stress relief	3.93	0.816
R3 Good mental state and increased concentration	4.06	0.766
R4 Physical health and increased level of activity	4.12	0.754
R5 Increased social interaction and reduced loneliness	3.96	0.806
R6 Improved sleep quality	4.04	0.783
**Visual sensation**		
V1 Variety of plants	4.23	0.694
V2 Richness of pants’ color	4.11	0.763
V3 Plant light and shadow mottle	4.01	0.789
V4 Nice road texture	4.00	0.841
V5 Rich terrain, wide view	4.24	0.732
V6 The water is highly ornamental	3.94	0.896
**Auditory sensation**		
A1 Sweet natural sound	4.14	0.787
A2 Sweet background music	3.80	0.908
A3 Sweet singing, instrumental sound	4.10	0.806
A4 Quiet space	4.10	0.913
A5 No traffic noise	4.03	0.854
**Tactile sensation**		
T1 The road material is comfortable and the foot feels good.	4.16	0.768
T2 People could get near to the water in the park and even touch it	3.72	1.047
T3 The seat is comfortable to sit up	4.10	0.813
T4 Comfortable grass for flat lay	3.99	0.944
**Behavioral activities in the park**		
B1 Relax and think (sit, read, and sun)	3.64	1.064
B2 Social interaction (chat, party, playing cards, dating)	3.26	1.243
B3 Fitness activities (fitness of fitness facilities, Tai Chi, dance, playing ball games)	3.08	1.386
B4 Literary activities (painting and calligraphy, musical instruments)	2.68	1.448
B5 Family activities (playing, taking children, walking the dog, taking pictures)	3.25	1.390
B6 Jogging, walking.	3.84	1.080
**Emotional responses**		
E1 Comfort (I feel comfort and peace)	4.26	0.651
E2 Pleasant feeling (I feel happy)	4.27	0.680
E3 Sense of belonging (I feel warm and belonged to this place)	3.98	0.801
E4 Attraction (I will come here again)	4.20	0.735

**Table 4:** Model fit test.

Fitting Index	Ideal Value	Acceptable Value	Model Predictive Value
Chi-sqr/DF	1–2	1–3	2.706
GFI	>0.9	>0.7	0.766
AGFI	>0.9	>0.7	0.725
RMSEA	<0.08	<0.09	0.082
TLI(NNFI)	>0.9	>0.7	0.781
CFI	>0.9	>0.7	0.801

**Table 5:** Structural equation model standardized path coefficient of observed variables.

Items	Standardized Path Coefficient
**Restorative effect**	
R1 Physical pain, not comfort	0.66
R2 Anxiety, depression, stress relief	0.63
R3 Good mental state and increased concentration	0.75
R4 Physical health and increased level of activity	0.74
R5 Increased social interaction and reduced loneliness	0.69
R6 Improved sleep quality	0.75
**Visual sensation**	
V1 Variety of plants	0.68
V2 Richness of pants’ color	0.68
V3 Plant light and shadow mottle	0.60
V4 Nice road texture	0.65
V5 Rich terrain, wide view	0.59
V6 The water is highly ornamental	0.60
**Auditory sensation**	
A1 Sweet natural sound	0.73
A2 Sweet background music	0.52
A3 Sweet singing, instrumental sound	0.48
A4 Quiet space	0.63
A5 No traffic noise	0.63
**Tactile sensation**	
T1 The road material is comfortable and the foot feels good.	0.63
T2 People could get near to the water in the park and even touch it	0.73
T3 The seat is comfortable to sit up	0.73
T4 Comfortable grass for flat lay	0.68
**Behavioral activities in the park**	
B1 Relax and think (sit, read, and sun)	0.60
B2 Social interaction (chat, party, playing cards, dating)	0.64
B3 Fitness activities (fitness of fitness facilities, Tai Chi, dance, playing ball games)	0.79
B4 Literary activities (painting and calligraphy, musical instruments)	0.56
B5 Family activities (playing, taking children, walking the dog, taking pictures)	0.59
B6 Jogging, walking.	0.75
**Emotional responses**	
E1 Comfort (I feel comfort and peace)	0.68
E2 Pleasant feeling (I feel happy)	0.81
E3 Sense of belonging (I feel warm and belonged to this place)	0.62
E4 Attraction (I will come here again)	0.71

**Table 6:** Results of path analysis: standardized estimates (N = 250).

Hypothesis	Coefficient	S.E.	*p*
Hypothesis 1 Restorative effect←Sensory perception			
Hypothesis 1a Restorative effect←Visual sensation	0.214	0.94	0.023 *
Hypothesis 1b Restorative effect←Auditory sensation	0.524	0.108	0.000 *
Hypothesis 1c Restorative effect←Tactile sensation	0.15	0.99	0.877
Hypothesis 2 Behavioral activities←Sensory perception			
Hypothesis 2a Behavioral activities←Visual sensation	0.269	0.121	0.027 *
Hypothesis 2b Behavioral activities←Auditory sensation	0.345	0.134	0.010 *
Hypothesis 2c Behavioral activities←Tactile sensation	0.454	0.139	0.001 *
Hypothesis 3 Emotional response←Sensory perception			
Hypothesis 3a Emotional response←Visual sensation	0.387	0.094	0.000 *
Hypothesis 3b Emotional response←Auditory sensation	0.254	0.096	0.008 *
Hypothesis 3c Emotional response←Tactile sensation	0.283	0.096	0.006 *
Hypothesis 4 Emotional response←Behavioral activities	0.149	0.064	0.020 *
Hypothesis 5 Restorative effect←Behavioral activities	0.191	0.063	0.003 *
Hypothesis 6 Restorative effect←Emotional response	0.277	0.096	0.004 *

Note: * *p* < 0.05.

**Table 7:** Results of mediation effect analysis (N = 250).

Variable	Effect	Point Estimate	Bootstrapping 95% Confidence Interval	
			Lower limit	Upper limit
Visual sensation→Restorative effect	Total effect	0.383	0.230	0.559
	Indirect effect	0.170	0.053	0.320
	Direct effect	0.214	0.046	0.405
Auditory sensation→Restorative effect	Total effect	0.674	0.436	0.959
	Indirect effect	0.150	0.063	0.299
	Direct effect	0.524	0.304	0.773
Tactile sensation→Restorative effect	Total effect	0.199	0.009	0.372
	Indirect effect	0.184	0.079	0.307
	Direct effect	0.015	−0.158	0.180
